# Combination of apigenin treatment with therapeutic HPV DNA vaccination generates enhanced therapeutic antitumor effects

**DOI:** 10.1186/1423-0127-16-49

**Published:** 2009-05-27

**Authors:** Chi-Mu Chuang, Archana Monie, Annie Wu, Chien-Fu Hung

**Affiliations:** 1 Department of Pathology, Johns Hopkins Medical Institutions, Baltimore, Maryland, USA; 2 Department of Oncology, Johns Hopkins Medical Institutions, Baltimore, Maryland, USA; 3 Department of Obstetrics and Gynecology, Taipei Veterans General Hospital, Taipei, Taiwan; 4 School of Medicine, National Yang-Ming University, Taipei, Taiwan

## Abstract

**Background:**

It is important to develop innovative therapies for advanced stage cancers in addition to the conventional therapies including chemotherapy, radiation and surgery. Antigen-specific immunotherapy has emerged as a novel alternate therapy for advanced stage cancers, which may be employed in conjunction with conventional therapies.

**Methods:**

In the current study, we tested the effect of treatment with the chemotherapeutic agent, apigenin in combination with DNA vaccines encoding the HPV-16 E7 antigen linked to heat shock protein 70 (HSP70) in the control of the E7-expressing tumor, TC-1.

**Results:**

We observed that treatment with apigenin rendered the TC-1 tumor cells more susceptible to lysis by E7-specific cytotoxic CD8+ T cells. Furthermore, treatment of TC-1 tumor cells with apigenin was found to enhance apoptotic tumor cell death in vitro in a dose-dependant manner. We showed that TC-1 tumor-bearing mice treated with apigenin combined with E7-HSP70 DNA generate highest frequency of primary and memory E7-specific CD8+ T cells, leading to potent therapeutic anti-tumor effects against E7-expressing tumors.

**Conclusion:**

Thus, apigenin represents a promising chemotherapeutic agent, which may be used in combination with immunotherapy for the treatment of advanced stage cancers. The clinical implications of the current strategy are discussed.

## Background

Advanced stage cancers are difficult to control using conventional therapies such as chemotherapy, radiation and/or surgery. Thus, the development of innovative therapies for advanced stage cancers is urgently needed. Antigen-specific immunotherapy has the potency to eradicate systemic tumors at multiple sites in the body, as well as the specificity to discriminate between malignant and normal cells. Thus, immunotherapy, such as DNA vaccination provides a promising alternate approach for advanced stage cancers, which may be employed in conjunction with conventional therapies.

DNA vaccination has the advantages of purity, simplicity of preparation, and stability. In addition, DNA-based vaccines can be prepared inexpensively and rapidly on a large scale (for review, see [1,2]). However, due to the low immunogenicity of DNA vaccines, strategies need to be developed to enhance DNA vaccine potency. Intradermal administration of DNA vaccines via gene gun represents an efficient way to deliver DNA vaccines into professional APCs in vivo. In addition, several strategies have been developed to enhance the potency of DNA vaccines by modification of the properties of antigen-presenting cells (APCs) (for review see [3-5]).

We have previously developed several intracellular targeting strategies to improve DNA vaccine potency by linkage of antigen to proteins that target the antigen for proteasomal degradation or entry into the endoplasmic reticulum (ER), thus facilitating MHC class I presentation of linked antigen in DCs. Vaccination with a fusion DNA construct encoding E7 antigen linked to *Mycobacterium tuberculosis *heat shock protein 70 (HSP70) (E7-HSP70) was shown to enhance E7-specific CD8+ T-cell-mediated immune responses and protect mice against E7-expressing TC-1 tumor growth [6]. This strategy is currently being translated to phase I/II clinical trials in patients with HPV-16 associated CIN lesions using the GMP grade E7-HSP70 DNA vaccine (pNGVL4a-Sig/E7(detox)/HSP70), which has been tested in human vaccine trials [7]. In addition, the same DNA vaccine has been used in HPV16+ patients with advanced head and neck squamous cell carcinoma (Dr. Maura Gillison, personal communication). Thus, the current DNA vaccine has significant potential for clinical translation for the treatment of HPV-associated malignancies.

Another innovative approach for the control of cancer is the employment of immunomodulatory chemotherapeutic agents such as flavonoids. Flavonoids, such as epigallocatechin gallate (EGCG)) have been shown to have positive immune modulating effects via mechanisms of antioxidant, antiinflammatory, and anti-cyclooxygenase activities [8,9]. Apigenin is of particular interest as an antitumor agent since it exhibits lower intrinsic toxicity and is not mutagenic as compared to other structurally related flavonoids [10]. Furthermore, apigenin has been shown to demonstrate antitumor effects in several tumor cell lines, including those derived from prostate, pancreatic and breast cancer [11-13]. Thus, apigenin represents a promising chemotherapeutic agent for cancer therapy.

In the current study, we aim to test the combination of apigenin treatment with E7-HSP70 DNA vaccination in the control of the E7-expressing tumor, TC-1. We observed that treatment with apigenin rendered the TC-1 tumor cells more susceptible to lysis by E7-specific CTLs. Furthermore, treatment of TC-1 tumor cells with apigenin was found to enhance apoptotic tumor cell death in vitro in a dose-dependant manner. We showed that TC-1 tumor-bearing mice treated with apigenin combined with E7-HSP70 DNA generate highest frequency of primary and memory E7-specific CD8+ T cells, leading to potent therapeutic anti-tumor effects against E7-expressing tumors. The clinical implications of the current strategy are discussed.

## Materials and methods

### Animals and Chemicals

Female C57BL/6 mice (H-2Kb and I-Ab), 5 to 6 weeks of age, were purchased from National Cancer Institute (Frederick, Maryland, USA) and kept in the oncology animal facility of the Johns Hopkins Hospital (Baltimore, Maryland, USA). Animals were used in compliance with institutional animal health care regulations, and all animal experimental procedures were approved by the Johns Hopkins Institutional Animal Care and Use Committee. Apigenin was purchased from Sigma-Aldrich (St. Louis, Missouri, USA).

### Murine Tumor Cell Line

The production and maintenance of TC-1 cells [14] and E7-specific T cells [15] have been described previously. Luciferase-expressing TC-1 cells (TC-1-luc) were generated by transducing TC-1 cells with retrovirus containing luciferase pLuci-thy1.1 and flow cytometry sorting following the protocol described previously. Briefly, In order to generate a retrovirus containing luciferase, a pLucithy1.1 construct expressing both luciferase and thy1.1 was made. Firefly luciferase was amplified by PCR from pGL3-basic (Promega) using the 5' primer CGGAGATCTATGGAAGACGCCAAAAAC and the 3' primer CGGGTTAACTTACGGCGATCTTTCC. The amplified luciferase cDNA was inserted into the *Bgl*II and *Hpa*I sites of the bicistronic vector pMIG-thy1.1. Both luciferase and thy1.1 cDNA are under the control of a single promoter element and separated by an internal ribosomal entry site (IRES). The pLuci-thy1.1 was transfected into Phoenix packaging cell line and the virion-containing supernatant was collected 48 h after transfection. The supernatant was immediately treated using a 0.45-mm cellulose acetate syringe filter (Nalgene, Rochester, New York, USA) and used to infect TC-1 in the presence of 8 mg/ml Polybrene (Sigma, St Louis, Missouri, USA). TC-1-luc cells were sorted using preparative flow cytometry of stained cells with thy1.1 antibody (BD, Franklin Lakes, New Jersey, USA). Both TC-1 cell line and TC-1-luc cell line were maintained in RPMI 1640, supplemented with 10% (v/v) fetal bovine serum, 50 U/ml penicillin/streptomycin, 2 mM L-glutamine, 1 mM sodium pyruvate, 2 mM nonessential amino acids, and 0.4 mg/ml G418 at 37°C with 5% CO_2_. On the day of tumor challenge, TC-1 cells were harvested by trypsinization, washed twice with 1× Hanks buffered salt solution, and finally resuspended in 1× Hanks buffered salt solution to the designated concentration.

### In Vitro Cytotoxicity Assay

Luciferase-expressing TC-1 cells [16] in medium were seeded into a 24-well round-bottom plate (1 × 10^5 ^cells/well). Twenty-four hours later, apigenin alone or in combination with E7-specific T cells were added into each well. Apigenin was dissolved in dimethyl sulfoxide (DMSO) and mixed with fresh medium to achieve the desired concentration of 0.2%. This concentration of DMSO did not alter cell growth [17]. Cells treated with solvent DMSO (0.1%) alone were used as controls. E7-specific cytotoxic T lymphocytes from the spleens of tumor bearing mice immunized with the DNA vaccine served as effector cells and were added in the amount of 1 × 10^6 ^cells/well. TC-1 cells expressing luciferase were used as target cells. After incubation, D-luciferin (potassium salt; Xenogen Corp.) was added to each well at 150 *μ*g/ml in media 5 min before imaging. Bioluminescence imaging was taken at baseline, 24, 48, and 72 hours using the IVIS Imaging System Series 200 (Xenogen, Cranbury, New Jersey, USA). An integration time of 10 s was used for luminescence image acquisition. CTL-mediated killing was assessed using bioluminescence-imaging systems quantifying the decrease of luminescence from baseline.

### Characterization of apoptotic cell death

The *in vitro *apoptotic effects of apigenin on TC-1 cells were evaluated using two-colored fluorescence using a phycoerythrin conjugated-Annexin V and 7-AAD apoptosis kit (BD PharMingen, San Diego, CA) following the manufacturer's instructions. Briefly, TC-1 cells were co-incubated with the indicated concentrations of apigenin, and DMSO (0.1% v/v) was used as negative controls. At 6 and 48 hr post-incubation, TC-1 cells were harvested and washed twice in cold PBS and resuspended in binding buffer at a concentration of 1 × 10^6^/ml. The cells were then exposed to the labeling solutions phycoerythrin conjugated-Annexin V and 7-AAD for 15 min. Flow cytometry analysis was performed using FACSCalibur with CELLQuest software (BD Biosciences, Mountain View, California, USA). Both early apoptotic (annexin V-positive and 7-AAD-negative) and late apoptotic (annexin V- negative and 7 AAD-positive) cells were included in the analysis.

### Plasmid DNA Constructs and Preparations

DNA fragment encoding *M. tuberculosis *Hsp70 was obtained from pKS70. For the generation of Hsp70-expressing plasmid (pcDNA3-hsp70), the hsp70 was subcloned from pKS70 plasmid into the unique *Bam*HI and *Hin*dIII cloning sites of the pcDNA3.1 expression vector (Invitrogen, Carlsbad, California, USA) downstream of the cytomegalovirus promoter [18]. The generation of HPV-16 E7-expressing plasmid (pcDNA3-E7) and the E7-hsp70 chimera DNA (pcDNA-E7-hsp70) has been described previously [6].

### DNA Vaccination

DNA-coated gold particles were prepared, and gene gun particle-mediated DNA vaccination was performed, according to a protocol described previously [6]. Gold particles coated with DNA vaccines (1 μg DNA/bullet) were delivered to the shaved abdominal regions of mice by using a helium-driven gene gun (Bio-Rad Laboratories Inc., Hercules, Calif.) with a discharge pressure of 400 lb/in^2^. C57BL/6 mice (5 per group) were immunized with 2 μg of the DNA vaccine and received a booster dose 7 days after the first vaccination.

### Intracytoplasmic Cytokine Staining and Flow Cytometry Analysis

Pooled splenocytes from mice treated with the various treatment regiments were harvested either 14 days (for primary immune response) and 42 days (for memory immune response) after tumor challenge. For the memory recall experiment, the vaccinated mice were re-vaccinated twice with E7-HSP70 DNA vaccine, 60 days after the last vaccination to generate the memory recall response. Ten days after the recall vaccination, splenocytes were harvested and incubated for 20 h with 1 μg/ml of E7 peptide containing an MHC class I epitope (aa49-57, RAHYNIVTF) in the presence of GolgiPlug (BD Pharmingen, San Diego, California, USA) [19]. The stimulated splenocytes were then washed once with FACScan buffer and stained with phycoerythrin-conjugated monoclonal rat anti-mouse CD8a (clone 53.6.7). Cells were subjected to intracellular cytokine staining using the Cytofix/Cytoperm kit according to the manufacturer's instruction (BD Pharmingen, San Diego, CA, USA). Intracellular IFN-γ was stained with FITC-conjugated rat anti-mouse IFN-γ. All antibodies were purchased from BD Pharmingen. Flow cytometry analysis was performed using FACSCalibur with CELLQuest software (BD Biosciences, Mountain View, California, USA).

### In Vivo Tumor Treatment Experiments

C57BL/6 mice were divided into four groups (control, apigenin only, E7-hsp70 only, and apigenin with E7-hsp70). All mice were inoculated with TC-1 cells (5 × 10^4^) s.c. over right leg. For the control group, mice were regularly followed after TC-1 implantation without specific treatment. For the apigenin only group, 3 days after TC-1 implantation, each mouse was given i.p. apigenin (25 mg/kg) and continued for 10 days. For the E7-hsp70 only group, 3 days after TC-1 implantation, each mouse was vaccinated with E7-hsp70 2 μg via gene gun first, and was boosted 7 d later. For the apigenin with E7-hsp70 group, after TC-1 implantation, each mouse received the same vaccination schedule as the E7-hsp70 only group, and the same i.p. apigenin schedule as the apigenin only group.

### Statistical Analysis

Statistical analysis was performed using Prism 3.0 software (GraphPad, San Diego, California, USA). Observed differences in apoptosis between different concentrations of apigenin were evaluated using student's *t *test. Differences in IFN-γ expression in CD8^+ ^cells were measured using Pearson's chi-square test. Survival curves were plotted using Kaplan-Meier method and compared by log-rank test. For all analyses *p *< 0.05 was considered statistically significant. Data are presented as mean ± standard deviation (SD) unless otherwise specified.

## Results

### Treatment with apigenin renders the TC-1 tumor cells more susceptible to lysis by E7-specific CTLs

**Figure 1:**
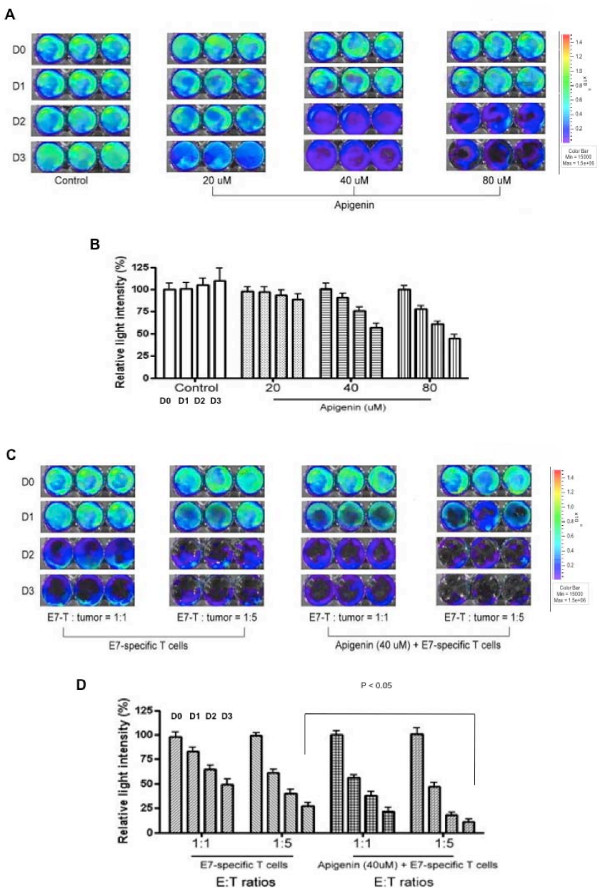
**In vitro cytotoxicity assay**. Luciferase-expressing TC-1 tumor cells were added to 24-well plates at a dose of 1 × 10^5^/well. TC-1/luc tumor cells were (A & B) treated with solvent DMSO (0.1%) as a control or treated with different concentrations of apigenin (20, 40, 80 μM) or (C & D) treated with E7-specific cytotoxic T cells at different E:T ratios (1:1, 1:5) with or without treated 40 mM apigenin. Bioluminescence imaging was performed on D0, D1, D2 and D3. The degree of CTL-mediated killing of the tumor cells was indicated by the decrease of luminescence activity using the IVIS luminescence imaging system series 200. Bioluminescence signals were acquired for 10 seconds. (A & C) Representative luminescence images of 24-well plates showing lysis of the tumor cells by (A) different concentrations of apigenin or by (C) E7-specific T cells and apigenin. (B & D) Bar graph depicting the quantification of luminescence intensity in tumor cells treated with (B) different concentrations of apigenin or treated with (D) apigenin and/or E7-specific cytotoxic T cells (mean ± SD). P values less than 0.05 are considered to be statistically significant. Data shown are representative of two experiments performed.

In order to determine if treatment of TC-1 tumor cells with apigenin will render the tumor cell more susceptible to E7-specific T cell-mediated killing, we performed a cytotoxicity assay using luciferase-expressing TC-1 tumor cells. TC-1/luc tumor cells were (i) treated with DMSO as a control, (ii, iii, iv) treated with different concentrations of apigenin, (v, vi) treated with E7-specific cytotoxic T cells at different E:T ratios (1:1, 1:5), or (vii, viii) treated with apigenin and E7-specific CTLs at different E:T ratios (1:1, 1:5). The CTL-mediated killing of the TC-1 tumor cells in each well was monitored using bioluminescent imaging systems over time. The degree of CTL-mediated killing of the tumor cells was indicated by the decrease of luminescence activity. As shown in Figure 1, cells treated with apigenin demonstrated a dose- and time-dependent reduction of luciferase activity. Furthermore, a significantly lower luciferase activity was observed in the wells incubated with apigenin and E7-specific CTLs at an E:T ratio of 1:5 as compared to the wells incubated with apigenin alone or E7-specific CTLs alone (p < 0.05). Thus, our data suggests that the TC-1 tumor cells treated with apigenin increased the susceptibility of the tumor cells for lysis by the E7-specific cytotoxic T cells.

### Treatment of TC-1 tumor cells with apigenin enhances the apoptotic tumor cell death in vitro in a dose-dependant manner

**Figure 2:**
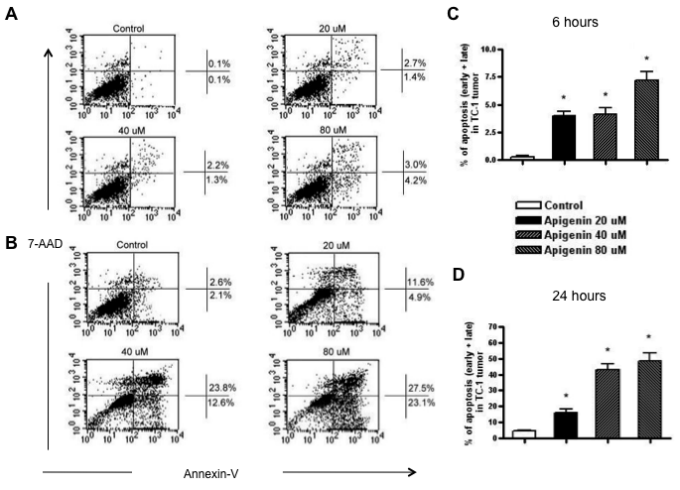
**Flow cytometry analysis to determine the number of apoptotic cells induced by apigenin treatment**. TC-1 cells were co-incubated with various concentrations of apigenin (20, 40, 80 μM). Cells treated with solvent DMSO (0.1%) alone were used as controls. The cells were then stained with PE-conjugated annexin V antibody (BD, San Diego) and 7-AAD to detect the expression of annexin V and 7-AAD. Flow cytometry analysis was performed to characterize the expression of annexin V+ 7-AAD+ (apoptotic) cells among the TC-1 cells treated with apigenin. A & B. Representative flow cytometry data demonstrating the percentage of apoptotic cells at (A) 6 hours after incubation or (B) 48 hours after incubation with different concentrations of apigenin. C & D. Bar graphs depicting in the percentage of apoptotic cells at (C) 6 hours after incubation or (D) 48 hours after incubation with different concentrations of apigenin. Data shown are representative of two experiments performed (mean ± SD).

In order to determine the effect of chemotherapy on TC-1 tumor cells, we incubated TC-1 tumor cells with different doses of apigenin (20, 40, 80 μM). The cells were then characterized for apoptotic cell death using annexin V and 7-AAD staining. As shown in Figure 2, we observed that TC-1 tumor cells treated with the higher doses of apigenin demonstrated a greater degree of apoptotic tumor cell death compared to control untreated TC-1 tumor cells both at 6 hours and at 48 hours following incubation with apigenin. Thus, our data indicate that treatment with apigenin enhances the apoptotic tumor cell death in TC-1 tumors in a dose dependant manner.

### TC-1 tumor-bearing mice treated with apigenin combined with E7-HSP70 DNA generate highest frequency of primary and memory E7-specific CD8+ T cells

**Figure 3:**
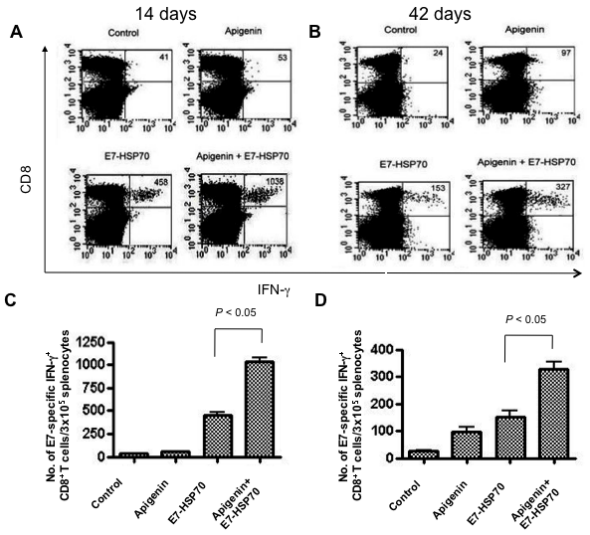
**Intracellular cytokine staining followed by flow cytometry analysis to determine the number of primary and memory E7-specific CD8^+ ^T cells in tumor-bearing mice treated with apigenin and/or E7-HSP70 DNA vaccine**. Groups of C57BL/6 mice (5 per group) were challenged subcutaneously with 1 × 10^4^/mouse of TC-1 tumor cells. Mice were treated with apigenin alone, E7-HSP70 DNA vaccine alone, the combination of apigenin and the E7-HSP70 DNA vaccine. Apigenin was administered intraperitoneally at the dose of 25 mg/kg after TC-1 inoculation and continued for 10 days. Mice were vaccinated with 2 μg/mouse of E7-HSP70 via gene gun, 3 days before TC-1 inoculation and receive a booster dose 7 days after the first vaccination. Untreated tumor challenged mice were used as negative controls. 14 days (for primary immune response) and 42 days (for memory immune response) after tumor challenge, splenocytes from mice were harvested and stained for CD8 and intracellular IFN-γ and then characterized for E7-specific CD8^+ ^T cells using intracellular IFN-*γ *staining followed by flow cytometry analysis. A & B. Representative data of intracellular cytokine stain followed by flow cytometry analysis showing the number of E7-specific IFNγ+ CD8+ T cells in mice treated with apigenin and/or DNA vaccine at (A) 14 days or (B) 42 days after tumor challenge. C & D. Bar graph depicting the numbers of E7-specific IFN-γ-secreting CD8^+ ^T cells per 3 × 10^5 ^pooled splenocytes at (C) 14 days or (D) 42 days after tumor challenge. Data shown are representative of two experiments performed (mean ± SD).

In order to determine the E7-specific CD8+ T cell immune response in tumor-bearing mice treated with apigenin in combination with the E7-HSP70 DNA vaccine, we first challenged groups of C57BL/6 mice (5 per group) with TC-1 tumor cells and then treated them with apigenin alone, E7-HSP70 DNA vaccine alone or apigenin in combination with DNA vaccination. Untreated tumor-bearing mice were used as negative controls. Splenocytes were harvested from vaccinated mice 14 days (for primary immune response) and 42 days (for memory immune response) after tumor challenge and characterized the presence of E7-specific CD8^+ ^T cells in treated mice using intracellular cytokine staining for IFN-γ followed by flow cytometry analysis. As shown in Figure 3, tumor-bearing mice that were treated with apigenin in combination with E7-HSP70 DNA generated a significantly higher number of primary as well as memory E7-specific CD8^+ ^T cells compared to tumor-bearing mice that were administered E7-HSP70 DNA alone or apigenin alone (*p *< 0.05). These results indicate that treatment of tumor-bearing mice with apigenin in combination with E7-HSP70 DNA leads to the strongest E7-specific CD8+ T cell immune responses.

### TC-1 tumor-bearing mice treated with apigenin combined with E7-HSP70 DNA generate a significant memory recall response of E7-specific CD8+ T cells

**Figure 4:**
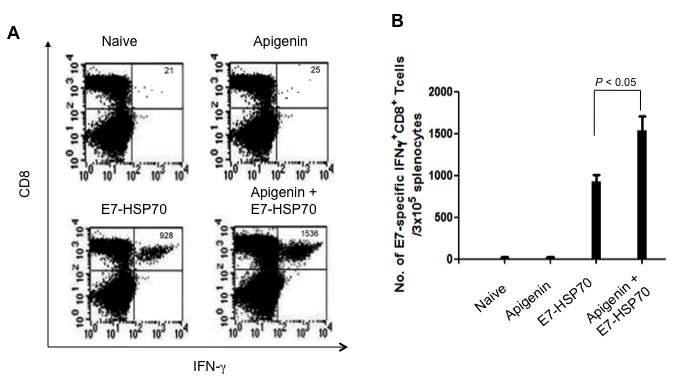
**Intracellular cytokine staining followed by flow cytometry analysis to determine the memory recall response of E7-specific CD8^+ ^T cells in tumor-bearing mice treated with apigenin and/or E7-HSP70 DNA vaccine**. Groups of C57BL/6 mice (5 per group) were challenged subcutaneously with 1 × 10^4^/mouse of TC-1 tumor cells. Mice were treated with apigenin alone, E7-HSP70 DNA vaccine alone, or the combination of apigenin and the E7-HSP70 DNA vaccine as described in Figure 3. Untreated tumor challenged mice were used as negative controls. Sixty days after the last vaccination, the mice were vaccinated twice with E7-HSP70 DNA vaccine to generate the memory recall response. Ten days after the recall vaccination, splenocytes were harvested and stained for CD8 and intracellular IFN-γ and then characterized for E7-specific CD8^+ ^T cells using intracellular IFN-*γ *staining followed by flow cytometry analysis. (A) Representative data of intracellular cytokine stain followed by flow cytometry analysis showing the memory recall response of E7-specific IFNγ+ CD8+ T cells in mice treated with apigenin and/or DNA vaccine. (B) Bar graph depicting the numbers of E7-specific IFN-γ-secreting CD8^+ ^T cells per 3 × 10^5 ^pooled splenocytes.

In order to determine the memory recall response of E7-specific CD8+ T cells in tumor-bearing mice treated with apigenin in combination with the E7-HSP70 DNA vaccine, we first challenged groups of C57BL/6 mice (5 per group) with TC-1 tumor cells and then treated them with E7-HSP70 DNA vaccine alone or apigenin in combination with DNA vaccination. Sixty days after the last vaccination, the mice were vaccinated twice with E7-HSP70 DNA vaccine to generate the memory recall response. Ten days after the recall vaccination, splenocytes were harvested and characterized for E7-specific CD8^+ ^T cells using intracellular IFN-*γ *staining followed by flow cytometry analysis. As shown in Figure 4, tumor-bearing mice that were treated with apigenin in combination with E7-HSP70 DNA generated a significant memory recall response of E7-specific CD8^+ ^T cells compared to tumor-bearing mice that were administered E7-HSP70 DNA alone or apigenin alone (*p *< 0.05). These results indicate that treatment of tumor-bearing mice with apigenin in combination with E7-HSP70 DNA leads to potent E7-specific CD8+ T cell memory recall responses.

### TC-1 tumor-bearing mice treated with apigenin combined with E7-HSP70 DNA vaccination generate the best therapeutic anti-tumor effects

**Figure 5:**
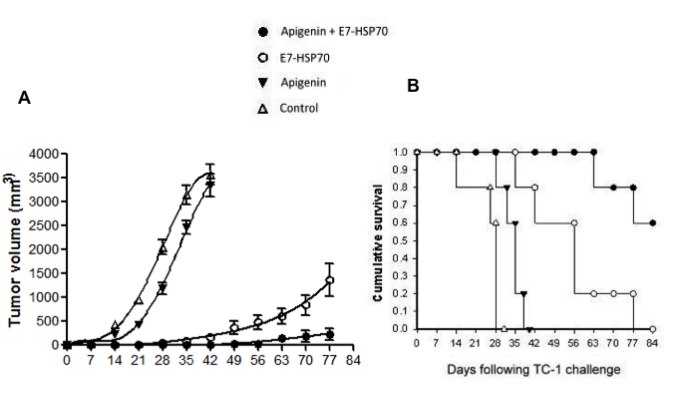
***In vivo *tumor treatment experiments**. Groups of C57BL/6 mice (5 per group) were subcutaneously challenged with 5 × 10^4^/mouse of TC-1 tumor cells. Tumor challenged mice were treated with apigenin and/or E7-HSP70 DNA vaccine as indicated in Figure 3. Untreated TC-1 tumor-bearing mice were used as a control. (A) Line graph depicting the tumor volume in TC-1 tumor bearing mice treated with apigenin and/or E7-HSP70 DNA. (B) Kaplan & Meier survival analysis of TC-1 tumor challenged mice treated with apigenin and/or E7-HSP70 DNA. Data shown are representative of two experiments performed (mean ± SD).

To determine the therapeutic antitumor effects generated by apigenin combined with E7-HSP70 DNA vaccination, we first challenged groups of C57BL/6 mice (5 per group) with TC-1 tumor cells and then treated them with apigenin alone, E7-HSP70 DNA vaccine alone or apigenin in combination with DNA vaccination. Untreated tumor-bearing mice were used as negative controls. As shown in Figure 5, tumor-bearing mice treated with apigenin in combination with E7-HSP70 DNA showed significantly reduced tumor size over time as compared to tumor-bearing mice treated with chemotherapy alone or the DNA vaccine alone (*p *= 0.001). Furthermore, tumor-bearing mice treated with apigenin in combination with E7-HSP70 DNA showed improved survival compared to tumor-bearing mice treated with apigenin alone or the DNA vaccine alone (*p *= 0.037). (Figure 1C). Thus, our data indicate that the treatment regimen using apigenin in combination with E7-HSP70 DNA vaccination produces the best therapeutic anti-tumor effects and long-term survival in TC-1 tumor-bearing mice.

## Discussion

In the current study, we observed that treatment with apigenin enhanced apoptotic tumor cell death and rendered the TC-1 tumor cells more susceptible to lysis by E7-specific CTLs. TC-1 tumor-bearing mice treated with apigenin combined with E7-HSP70 DNA were found to generate significant effector and memory E7-specific CD8+ T cell immune responses, thus generating strong therapeutic anti-tumor effects. Thus, treatment with the combination of DNA vaccine and apigenin is efficient in generated significant antitumor effects against E7-specific tumors.

In our study, we observed that treatment with apigenin led to an increase in the apoptotic cell death of the TC-1 tumor cells (See Figure 2). Our results are consistent with previous studies by other investigators. For example, apigenin has previously been shown to inhibit the growth of human cervical carcinoma cells by inducing apoptosis through a p53-dependant pathway by Zheng *et al *[20]. They also showed that the HeLa cells treated with apigenin were arrested at G1 phase and demonstrated increased expression of the pro-apoptotic factors, Fas/APO-1, caspase-3 and p21/WAF1 protein and decreased expression of the anti-apoptotic protein, Bcl-2. Thus, the employment of apigenin may lead to apoptotic cell death in a variety of tumors.

The increased apoptotic tumor cell death caused by treatment with apigenin may contribute to the observed enhancement in antigen-specific immune responses generated by DNA vaccination (See Figure 3). One potential mechanism for the observed effect is that the apoptotic tumor cells may be taken up by antigen-presenting cells, resulting in the activation of tumor-specific CD8+ T cells (so called cross-priming mechanism). We and others have previously shown that chemotherapeutic agents that are capable of causing apoptotic cell death such as EGCG [21], cisplatin [22] or bortezomib [23] can significantly enhance the HPV antigen-specific CD8+ T cell immune responses induced by therapeutic HPV DNA vaccination, resulting in potent antitumor effects. Therefore, the apoptotic cell death caused by apigenin may contribute to the observed enhancement in the DNA vaccine potency.

For clinical translation, it is important to address issues regarding drug toxicity. Apigenin has been tested in several clinical trials and proven to be safe in humans [24,25]. Furthermore, it is also important to address concerns regarding the potential for oncogenicity associated with administration of E7 as DNA vaccines into the body. Thus, the clinical grade GMP grade E7/HSP70 DNA vaccine that is being used for our clinical studies (pNGVL4a-Sig/E7(detox)/HSP70) [7] encodes attenuated (detox) versions of E7 antigen that has a mutation at position 24 and/or 26, which will disrupt the Rb binding site of E7, abolishing the capacity of E7 to transform cells [26]. Furthermore, we have also used codon optimized E7 DNA in our vaccine constructs, which has been shown to enhance the expression of E7 antigen in DCs, leading to increased translation of the DNA vaccine in DCs [27,28].

In addition, clinical translation may preclude the use of the pcDNA3 vector, because it contains an ampicillin resistance gene. Thus, our clinical grade DNA vaccine has used the pNGVL4a vector obtained from the NIH National Gene Vector Laboratory for DNA vaccine development. This vector is a second-generation plasmid derived from pNGVL-3, which has been previously used for human clinical trials [29]. The pNGVL4a vector lacks the ampicillin resistance gene and thus would be suitable for clinical translation of the current DNA vaccines.

In summary, our data suggest that vaccination with E7-HSP70 DNA vaccine in combination with apigenin generates significantly better E7-specific immune responses as well as therapeutic antitumor effects. The preclinical observations serve as an important foundation for future clinical translation using apigenin in combination with therapeutic HPV DNA vaccines for the control of HPV-associated lesions.

## Competing interests

The authors declare that they have no competing interests.

## Authors' contributions

CMC was involved in the execution of the project. AM and AW were involved in the interpretation of the data and writing the manuscript. CFH provided overall supervision and guidance for the project. All authors read and approved the manuscript.
